# Assessment of prevalence and risk factors of isolated coronary artery ectasia: A 5-year double-center retrospective study in Yazd, Iran 

**DOI:** 10.22088/cjim.15.2.244

**Published:** 2024

**Authors:** Hossein Nough, Fatemeh Moradi, Hamid Reza Varasteravan, Leila Afkhami, Marzieh Azimizadeh, Hamidreza Mohammadi, Mohammad Shafiee, Mahmood Emami, Naser Hossein Sartipzade, Farzan Safi Dahaj, Arman Nough

**Affiliations:** 1 Yazd Cardiovascular Research Center, Non-communicable Diseases Research Institute, Shahid Sadoughi University of Medical Sciences, Yazd, Iran; 2 Shahid Sadoughi University of Medical Sciences, Yazd, Iran; 3 Department of Cardiology, Shahid Sadoughi Hospital, Shahid Sadoughi University of Medical Sciences, Yazd, Iran; 4 Department of Radiology, Shariati Complex Hospital, Tehran University of Medical Sciences, Tehran, Iran; 5 Student Research Committee, Faculty of Medicine, Shahid Sadoughi University of Medical Sciences, Yazd, Iran

**Keywords:** Isolated coronary artery ectasia, Coronary artery disease, Coronary angiography, Markis, Harikrishnan.

## Abstract

**Background:**

The prevalence of Coronary artery ectasia (CAE) varies from 0.‌3 to 5% in different countries. The prevalence of CAE has varied in different parts of the world and the study of risk factors can be effective in the process of diagnosis and treatment of patients, we reviewed patients who underwent coronary angiography for 5 years to determine the prevalence of isolated CAE and its associated risk factors.

**Methods:**

A retrospective analysis was conducted on 16600 patients who underwent coronary angiography at Shahid Sadoughi and Afshar hospitals between March 2015 to April 2020. Diagnosis and confirmation of CAE was defined as a vessel diameter greater than 1.5 times the normal diameter of the vessel, which must be confirmed by at least two cardiologists. Demographic variables, angiography and echocardiography reports were included in our final analysis.

**Results:**

Isolated CAE was diagnosed in 287 (1.7%) patients. After triple-vessel disease (53%), the left anterior descending artery (LAD) was the commonest affected vessel by ectasia 16% (46 cases). Diffuse isolated CAE was diagnosed in 52% of LAD, 76.6% of Right coronary artery (RCA), and 74.1% of left circumflex artery. A significant association was seen between the vessel involved and the nature of ectasia (p<0.001).

**Conclusion:**

In our study, the occurrence of isolated CAE was similar to other studies. This condition often affects all three major vessels of the coronary arteries, and is commonly categorized as type 1, which involves diffuse involvement of the arteries based on the Markis and Harikrishnan Classification.

**C**oronary artery disease (CAD) is one of the most common cardiovascular diseases (1). Coronary artery ectasia (CAE) is defined as diffuse or localized dilation 1.5 times the normal portion of an adjacent vessel and is a type of CAD (2). The prevalence of CAE varies from 0.‌3% to 5% across different countries (3, 4). Usually, coronary angiography is used for the Diagnosis of CAE. However, the exact cause of CAE has not been definitively determined, but it is believed to be associated with a variety of factors, including degenerative, congenital, inflammatory, infectious, and traumatic causes (5). 

Ectasia is commonly associated with atherosclerosis or may occur as a compensatory mechanism in cases with proximal stenosis in the anterior coronary artery (6, 7). Moreover, it is also seen in pulmonary artery abnormalities or high-flow conditions such as a coronary artery fistula (8). Although male gender, history of hypertension, and smoking are more common in patients with CAE (9), the patients with diabetes have a 

lower risk of developing to CAE due to the their low regulation of MMP, which is associated with negative regeneration in response to atherosclerosis (10, 11).

Due to the prevalence of CAE has varied in different regions, understanding its associated risk factors can facilitate the diagnosis and treatment of patients. Therefore, we conducted a retrospective study of patients who underwent coronary angiography over a five-year period to identify the prevalence of isolated CAE and its potential risk factors.

## Methods

This is a retrospective study conducted at Shahid Sadoughi and Afshar hospitals in Yazd, Iran, between March 2015 to April 2020. The study was approved by the Ethics Committee of Shahid Sadoughi University of Medical Sciences, Yazd, and the need for patient written informed consent was waived (IR.SSU.MEDICINE.REC.1399.174).

The study included 16,600 patients who underwent coronary angiography, and all patients were evaluated for hematologic and laboratory tests, including renal function test for contrast-induced nephropathy after coronary angiography. Patients with a previous history of open-heart surgery, a history of coronary stenting, and patients with moderate & severe CAD were excluded.

Coronary angiography reports were inspected, and a reevaluation of coronary angiography film was done by one different interventionist. Diagnosis and confirmation of CAE were defined as a vessel diameter greater than 1.5 times the normal diameter of the vessel, which must be confirmed by at least two cardiologists (2). CAE was classified according to the Markis et al. classification (12) and Harikrishnan et al. classification (13). Isolated CAE was defined as ectasia without moderate & severe CAD (14). The cardiologists evaluated the angiographic reports of all patients and identified cases of isolated CAE.

Demographic variables includes age, sex, body mass index (BMI), smoking, and medical conditions such as diabetes mellitus (DM), hypertension (HTN), and hyperlipidemia (HLP), along with angiography and echocardiography report, including Left ventricular ejection fraction (LVEF), the number of vessels involved, and the type of vascular involvement (localize, diffuse) were collected from catheterization films and medical records. 

The data were analyzed using the SPSS 22 for Windows statistical package. Continuous variables were presented as means and standard deviations, while categorical variables were presented as numbers and percentages, and compared using the Chi-Square test. A p-value of less than 0.05 was considered statistically significant.

## Results

During the five years, in two hundred and eighty-seven patients (1.7%), isolated CAE was diagnosed. The mean age was 52.26 ± 12 years (range 27 to 85years). About 126 (43.9%) were overweight (BMI greater than 25 kg/m^2^) (15), and 71 (24.7%) had DM. We show other demographic variables in the table (Table 1). 

**Table 1:** Baseline clinical characteristics

**Variable**	**Frequency**	**Percentage**
**Sex, Male**	165	57.5
**Smoking**	69	24
**Diabetes mellitus**	71	24.7
**Hypertension**	127	44.2
**Hyperlipidemia**	88	30.6
**Overweight**	126	43.9

Coronary angiography showed a single-vessel disease in 55 cases (19.1%), double-vessel disease in 80 cases (27.9%), and triple-vessel disease in 152 cases (53%). After triple-vessel disease (53%), the left anterior descending artery (LAD) was the commonest affected vessel by ectasia 16% (46 cases). Echocardiography results show that 131 (45.6%) patients had normal LVEF (Table 2). 

**Table 2:** The distribution of lesion vessels and LVEF in patients with coronary artery ectasia

**Variable**		**Frequency**	**Percentage**
***LVEF***			
**sever LV dysfunction, EF<30**		13	4.5
**moderate LV dysfunction, 35-40**		18	6.3
**mild LV dysfunction , 45**		27	9.4
**Preserved , 45-50**		98	34.1
**Normal, above 50%**		131	45.6
***Lesion vessels***			
**single-vessel**	**LAD**	46	16
	**RCA**	5	1.7
	**LCX**	4	1.4
**double-vessel**	**LAD+LCX**	37	12.9
	**LAD+RCA**	39	13.6
	**LCX+RCA**	4	1.4
**triple-vessel**	**LAD+RCA+LCX**	152	53

LAD, left anterior descending artery. RCA, right coronary artery. LCX, left circumflex coronary artery

**Table 3:** Angiographic types according to Markis et al. Classification

**Type**	**Ectasia type and location**	**Isolated coronary artery ectasia (n=287)**
**Type I**	Diffuse ectasia of two or three vessels	143 (49.8%)
**Type II**	Diffuse disease in one vessel and localized disease in another vessel	70 (24.4%)
**Type III**	Diffuse disease in one vessel only	11 (3.8%)
**Type IV**	Localized or segmental ectasia	63 (22%)

**Table 4:** Angiographic types according to Harikrishnan et al. Classification

**Type**		**Description**	**Isolated coronary artery ectasia** **(n=287)**
**Type I**	**Ia**	Diffuse ectasia in three vessels	82 (28.6%)
	**Ib**	Diffuse ectasia in two vessels and a localized ectasia in another vessel	40 (13.9%)
	**Ic**	Diffuse ectasia in two vessels	25 (8.7%)
**Type II**	**IIa**	Diffuse ectasia in one vessels and localized in another vessel	44 (15.3%)
	**IIb**	Diffuse ectasia in one vessels and localized in other two vessels	31 (10.8%)
**Type III**		Diffuse ectasia in one vessel	2 (0.7%)
**Type IV**	**IVa**	One vessel involved	41 (14.3%)
	**IVb**	Two vessels involved	14 (4.8%
	**IVc**	Three vessels involved	8 (2.8%)

CAE extension was assessed according to the classification proposed by Markis et al: 49.8% patients were classified 49.8% as type I; 24.4% as type II; 3.8% as type III and 22% as type IV (Table 3). Also according to Harikrishnan et al. Classification: 28.6% patients were classified as type Ia (Table 4). According to Markis et al. classification, diffuse isolated CAE was diagnosed in 52% of LAD, 76.6% of right coronary artery (RCA), and 74.1% of left circumflex artery (LCX). A significant association was seen between the vessel involved and the nature of ectasia (p<0.001) (table 5). 

The distribution of vessels involved as per the modified Markis classification and its relation to coronary risk factors was given in table 6. Comparing the four different subgroups of CAE, There were no statistical differences in DM, hyperlipidemia, hypertension, and overweight (p>0.05), but there were statistically differences in sex (P=0.013) (table 6). 

**Table 5:** Nature of Ectasia and vessel distribution

**Vessel**	**Focal** **N= 229**	**Diffuse** **N= 440**	**P-value**
**LAD**	132 (48)	143 (52)	<0.001
**RCA**	46 (23.4)	151 (76.6)	
**LCX**	51 (25.9)	146 (74.1)	

LAD, left anterior descending artery. RCA, right coronary artery. LCX, left circumflex coronary artery

**Table 6:** Modified Markis Classification et al. and Coronary Risk Factor

**Coronary risk factors**	**Markis Classification**				**P-value**
	**Type I**	**Type II**	**Type III**	**Type IV**	
**Sex, male**	50 (35)	29 (41.4)	6 (54.5)	37 (58.7)	0.013
**Diabetes mellitus**	36 (25.2)	15 (21.4)	2 (18.2)	18 (28.6)	0.86
**Hypertension**	68 (47.6)	31 (44.3)	5 (45.5)	23 (36.5)	0.66
**Hyperlipidemia**	44 (30.8)	19 (27.1)	6 (54.5)	19 (30.2)	0.32
**Smoking**	31 (21.7)	16 (22.9)	3 (27.3)	19 (30.2)	0.23
**Overweight**	51 (35.7)	36 (50)	5 (55.6)	33 (52.4)	0.06

## Discussion

CAE is an infrequently observed vascular phenotype in patients undergoing coronary angiography (16-18). However, there remains a lack of well-defined understanding regarding the etiology, clinical significance, risk factors, and treatment options for CAE (19), and various hypotheses have been proposed. In a study by Celik et al., it was demonstrated that atherosclerosis may play a role in the pathogenesis of isolated CAE, given the observed increase in carotid intima-media-thickness in patients with isolated CAE (20).

The incidence of CAE as detected by coronary arteriogram has been observed to range from 0.3% to 5% (3). In India, three studies on CAE reported a prevalence of 3.9%, 10.2%, and 5.6%, respectively.(21-23). A survey of the nations of the Middle East showed that the prevalence of CAE in studies from Iran, Turkey, and Saudi Arabia were 2.3% (24), 1.38% (25), and 6% (26), respectively. In China, the prevalence of CAE was 0.6% (27). The European studies showed that the prevalence of CAE in London, Greece, and Spain were 1.4% (18) , 3.1% (28), and 3.3% (29), respectively. In the USA's largest series from the Coronary Artery Surgery Study (CASS) registry, Swaye et al. found CAE in 4.9% of coronary angiograms (30). The prevalence of ectasia ranges from 0.6 to 12% in different countries, and it seems that race and genetic differences can affect CAE. In addition, the prevalence of CAE varies in a country with slightly different racial and genetic differences, indicating demographic and lifestyle variables contribute to the prevalence of CAE. It is noteworthy that the patients in this study referred to Shahid Sadoughi and Afshar hospitals from 6 provinces of Iran (Fars, Kerman, Hormozgan, Sistan and Baluchestan, South Khorasan, and Yazd), and those who had different living conditions and genetic. The emergence of new noninvasive technologies, such as computed tomography (31), magnetic resonance (24), and coronary angiography, may lead to an increased recognition rate of CAE. For instance, Zeina et al. reported a prevalence rate of 8% for CAE detected via coronary CT angiography (32).

Apart from the CASS in which 20,087 patients were studied, no such large series have been published (30) While in the present study, among 16,600 patients who underwent coronary angiography, isolated CAE was diagnosed in 287 cases (1.7%) in Shahid Sadoughi and Afshar hospitals. In the absence of coronary stenosis and other heart diseases, isolated CAE is rare; according to Al-Harthi et al. noted, the prevalence of isolated CAE was 0.1–0.79% (33). Malviya et al. found that the prevalence of isolated CAE was 1.05% (34). In Sultana et al.’s study, isolated CAE with no obstructive coronary lesions was seen in 75(1.5%)patients. (35). Debate persists regarding the risk factors for CAE, and some studies indicating an association between CAE and traditional cardiovascular risk factors (36). In George et al.’s study, CAE was significantly predominant in men compared with women (16). More studies have reported that CAE frequency in males was higher than females (36). In the present study, 57.5% of patients with isolated CAE were males was generally consistent with Aksu et al.’s study in which the male gender was demonstrated as an independent predictor of CAE (37). In the previous study, the mean age of patients was 53.4 years (34). In our study, the mean age of the patients was 56.26 years. Age-related factors such as degenerative changes, inflammatory diseases, and atherosclerosis are influential in the etiology of CAE (35). Boles et al. identified smoking as a potential risk factor for CAE(38). This finding is supported by the fact that smoking can cause injury to vascular endothelium, which in turn could lead to atherosclerosis as a possible pathophysiological mechanism (39). In our study, 24% of patients with isolated CAE had a smoking history; more studies need to clarify this association. Some studies have reported that HTN and HLP were significantly correlated with CAE (40). In a Harikrishnan et al.’s study, the prevalence of HTN, DM, and HLP in patients with CAE were 45.4%, 27.2%, and 90.9%, respectively (13). In the present study, the frequency of DM, HTN, and HLP in isolated CAE were 24.7%, 44.3%, and 30.7%, respectively. We found that the vessels involved as per the modified Markis classification and its relation to coronary risk factors show no statistical differences, but there were statistically differences in sex. Unlike other studies, fifty-three percent of isolated CAE subjects showed triple-vessel involvement in the present study. The involvement of coronary vessels in CAE is known to vary, with several studies indicating that RCA is the most commonly affected vessel, while other studies suggest that the LAD is the main vessel involved (34, 35, 41). We found that LAD was the most commonly affected vessel, followed by RCA and the LCX. According to Markis classification, 49.8% of patients were classified as type I (12). However, Almansori et al. noted that type IV was the most common involvement (26), and Takahito et al. found that type I was the most common presentation noted, followed by type III (41). Demopoulos et al. and Harikrishnan et al. found the ectasia of diffuse nature was predominantly seen in RCA with LAD (13, 42). Malviya et al. noted that ectasia of diffuse nature was predominantly seen in RCA (34). In the present study, diffuse ectasia's frequency was significantly higher; in RCA and LCX, diffuse ectasia was more than 70%. Overall, it can be concluded that vascular involvement in isolated CAE was often diffuse type. This finding is in agreement with earlier research that reported diffuse disease and involvement of multiple vessels (12, 43, 44). However, this study has a few limitations. Firstly, there was a lack of patients who were followed up and had angiographic follow-up. Secondly, there was no appropriate control group available for comparison. Thirdly, data on patients' signs and symptoms and medications taken by patients was not available.

The prevalence of isolated CAE in our study was close to other studies, and often affects all three major vessels of the coronary arteries and was more of the type 1 that with diffuse involvement of the arteries. The isolated CAE was also more common in middle-aged men, and there was a relationship between the frequency distribution of sex and the vessel involved according to Markis classification.
